# An In Vivo Microbial Assessment of Cotton, Polytetrafluoroethylene (PTFE) Tape, and Endo Foam As Spacer Materials Combined With Intracanal Medicaments in Endodontic Treatment

**DOI:** 10.7759/cureus.84559

**Published:** 2025-05-21

**Authors:** Manchala SaiKrishna, Divya Harika Pedada, Amit Raj Kantha, Pundari Deveneni, Ravalika K, Shruti Agarwal

**Affiliations:** 1 Department of Conservative Dentistry and Endodontics, Meghna Institute of Dental Sciences, Nizamabad, IND; 2 Department of Oral Medicine and Radiology, Panineeya Institute of Dental Sciences and Research Centre, Hyderabad, IND; 3 Department of Conservative Dentistry and Endodontics, Sri Sai College of Dental Surgery, Vikarabad, IND; 4 Department of Conservative Dentistry and Endodontics, MNR Dental College and Hospital, Hyderabad, IND

**Keywords:** cavit w, endo foam, intracanal medicaments, polytetrafluoroethylene (ptfe), root canal therapy

## Abstract

Background: To evaluate the efficiency of cotton (Apollo Sterilized Cotton Balls, Apollo Hospitals Enterprise Limited, Chennai, India), polytetrafluoroethylene (PTFE) tape (Holdtite Plus PTFE Tape, Pidilite Industries, Mumbai, India), and endo foam (Super Endo, Shenzhen Superline Technology Co., Ltd., Shenzhen, China) as an endodontic spacer material in combination with different intracanal medicaments.

Materials and methods: Ninety patients were randomized into three groups: Group I: cotton (n = 30), Group II: PTFE tape (n = 30), and Group III: endo foam (n = 30) after access opening and biomechanical preparation. Each group was further subdivided based on the intracanal medicament into subgroup A: sterile spacer (n = 10), subgroup B: spacer + calcium hydroxide (Ca(OH)_2_) (Prime Dental Products Pvt. Ltd., Thane, India) (n = 10), and subgroup C: spacer + modified triple antibiotic paste (MTAP) (n = 10). Microbial load was assessed from samples collected from the access cavity at baseline (S_1_) and again after seven days (S_2_). Colony-forming units (CFUs) were determined after a 48-hour aerobic culture on brain-heart infusion (BHI) agar.

Results: All three groups showed a statistically significant difference between the baseline and after seven-day mean values (p = 0.045, p = 0.049, p = 0.047). Intergroup comparison revealed a statistically significant difference in the mean values between the cotton and PTFE tape groups, as well as between the PTFE tape and endo foam groups. The mean difference values between the cotton and endo foam groups, however, did not differ in a way that was statistically significant.

Conclusion: Within the limitations of the study, it can be concluded that the PTFE tape and endo foam groups performed better than cotton.

## Introduction

Microbial infection is the leading cause of endodontic infections, making root canal therapy essential for healing and ongoing maintenance of the periradicular tissue [1]. A well-established fact in endodontics is that the integrity of the coronal seal is as essential as the root canal filling [2]. This remains applicable even in the case of multi-visit endodontic treatments. Provisional restorations are required when a multiple-visit strategy is chosen and when a definitive coronal restoration is to be performed during a succeeding appointment [3]. Coronal leakage through provisional restorations can complicate endodontic therapy between appointments. Previous investigations have revealed that the temporary restorative material thickness is a key factor in minimizing the leakage into the canal system [4]. Webber et al. showed that a minimum thickness of 3-4 mm of Cavit is required to ensure an effective seal [5]. 

To avoid undesirable materials from obstructing the canal space, a "spacer" or "barrier material" should be placed apically to the provisional restoration [3]. It allows easy removal, shorter access time to canals, and reduced tooth damage [6]. Cotton is the most commonly used endodontic spacer. Recently, other materials like polytetrafluoroethylene (PTFE) tape and endo foam have also been used. The ideal requirements of spacer materials should be easy to handle, cost-effective, easy to place and remove, visible, autoclavable, inert, have a restricted volume, support provisional restoration, and have minimal or no leakage [3].

Cotton is a common spacer beneath temporary restorative materials, but it can compromise the seal by reducing restoration thickness, leading to microbial leakage. Its fibers may incorporate into the restorative material, adhere to cavity walls, and act as a wick, affecting stability and cement adaptation [7]. Due to the following reasons, clinicians now employ foam pellets and PTFE tape as spacers. PTFE tape is a flexible material that is widely used in dentistry these days. It is an inorganic, non-fibrous, ribbon-like material and minimizes the potential for bacterial uptake by wicking [8]. Endodontic foams, made of biocompatible polyurethane, offer chairside storage and mechanical cleaning and serve as compressible, resilient spacers in endodontic procedures [1]. RC Cal (Prime Dental Products Pvt. Ltd., Thane, India) is a ready-to-use paste containing calcium hydroxide (Ca(OH)₂) and barium sulfate (BaSO₄), known for its effectiveness in various dental applications. It is highly alkaline, radiopaque, and water-soluble, allowing for easy cleaning and extraction from the canal when needed [9]. Triple antibiotic paste (ciprofloxacin, metronidazole, and minocycline) has been modified with clindamycin to prevent staining [10]. Cavit™ W (3M, Maplewood, MI, USA) is a self-curing temporary sealing material used for cavity restoration, offering quick, void-free curing in a moist environment. Its slight expansion ensures a well-sealed margin [11]. This study focuses on evaluating materials like cotton, PTFE tape, and endo foam for their role as spacers in combination with intracanal medicaments.

## Materials and methods

Study design

This study was approved by the Institutional Ethical Committee of Meghna Institute of Dental Sciences, Nizamabad, India (approval number MIDS/MDS/CONS/009). A power analysis using G*Power statistical software (Ver. 3.1, Heinrich-Heine-Universität Düsseldorf, Düsseldorf, Germany) (α = 0.05; power = 0.80) determined that 90 samples were required.

Inclusion and exclusion criteria

Ninety human mandibular molars from patients aged between 20 and 40 years, with intact walls and diagnosed with symptomatic or asymptomatic irreversible pulpitis and apical periodontitis, were included. Exclusions comprised patients with abscesses, cysts, perforations, developmental anomalies, immunocompromised patients, and pregnancy. Patients who had received antibiotic therapy, including prophylaxis for extractions, within the past three months were excluded from the study to prevent alterations in the oral microbial flora. Additionally, all restorations were examined for marginal integrity and leakage prior to sample collection. Any teeth with fractured, loose, or dislodged restorations at baseline were excluded, and any such changes during the study period were recorded; those cases were excluded from the final analysis.

Procedure

Informed consent was obtained, and a local anesthetic agent, Lignox 2% (Indoco Remedies Ltd., Goa, India), was administered. A new sterile Endo Access #3 bur (Dentsply Maillefer, Charlotte, NC, USA), along with a handpiece, was used to prepare an access cavity under rubber dam isolation. In a Class II cavity, rubber dam isolation was done after pre-endodontic build-up using composite, with rubber dam seal material utilized in the event of a leak. An electronic apex locator (Root ZX mini, J. Morita Corp., Kyoto, Japan) was used to establish the working length, and a radiograph was used for confirmation. Sodium hypochlorite (NaOCl) and saline were used for extensive irrigation. Biomechanical preparation was done using a crown-down technique using ProTaper Gold (Dentsply Sirona, Charlotte, NC, USA) up to F2. After biomechanical preparation, the canals were irrigated with 17% ethylenediaminetetraacetic acid (EDTA) for one minute, followed by a final saline rinse.

Molars were randomly assigned to three groups based on spacer material: Group I included cotton spacers (Apollo Sterilized Cotton Balls, Apollo Hospitals Enterprise Limited, Chennai, India) (n = 30), Group II consisted of PTFE tape spacers (Holdtite Plus PTFE Tape, Pidilite Industries, Mumbai, India) (n = 30), and Group III comprised endo foam spacers (Super Endo, Shenzhen Superline Technology Co., Ltd., Shenzhen, China) (n = 30). Each group was further divided into three subgroups based on medicament: Subgroup A: Sterile spacer (n = 10), Subgroup B: Spacer + Ca(OH)₂ paste (n = 10), Subgroup C: Spacer + Modified triple antibiotic paste (MTAP) (n = 10). The PTFE tape was standardized to a length of approximately 2.5 inches.

**Figure 1:**
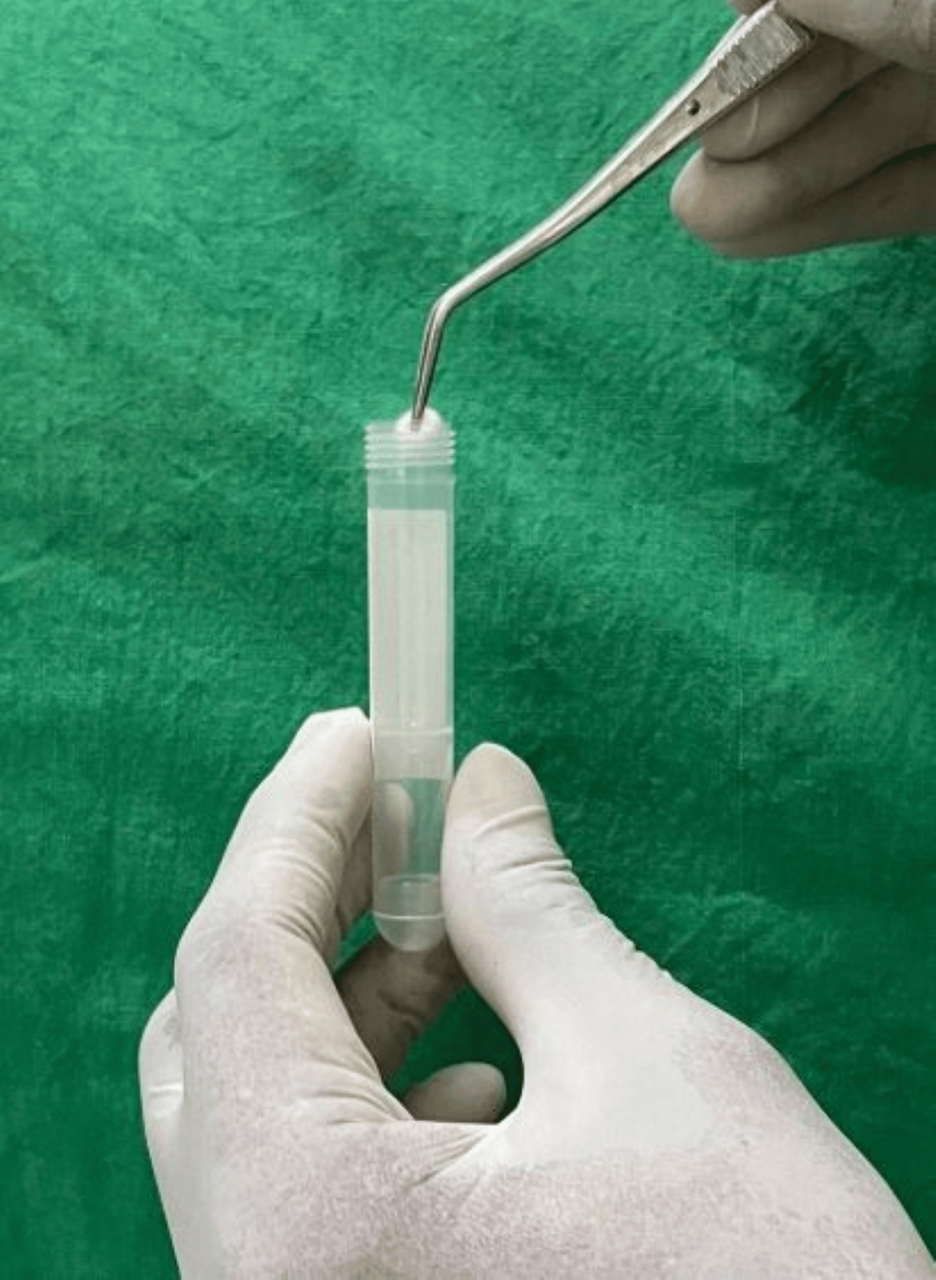
Collection of the sample

**Figure 2:**
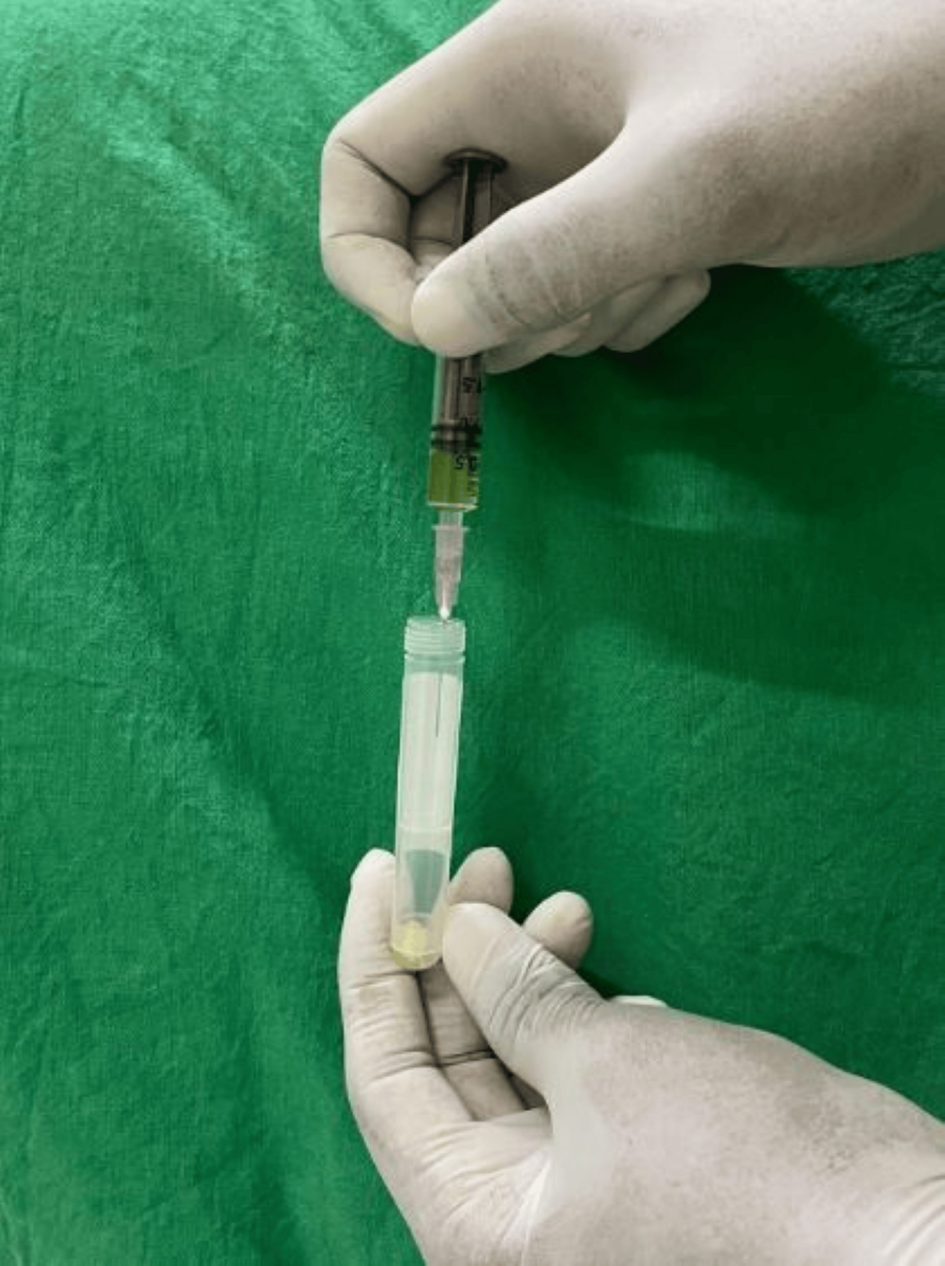
Introduction of brain heart infusion (BHI) broth into the container with the sample

Baseline sample (S₁) was collected in a container (Figure 1), and brain heart infusion broth (BHI broth) (HiMedia Laboratories, Thane, India) was introduced (Figure 2). Spacer was inserted into the access cavity according to the respective groups and subgroups and restored with Cavit™ W of 3 mm thickness. After one week, patients were recalled, and rubber dam isolation was performed. A second sample (S₂) was collected after removing the Cavit™ W and spacer. The tubes were then incubated at 37° C for 48 hours. All the samples were cultured in BHI agar plates, and colony-forming units (CFUs) were calculated.

Statistical analysis

The statistical analysis was performed using IBM SPSS Statistics software, version 25.0 (IBM Corp., Armonk, NY). The resultant values were statistically analyzed using the one-way ANOVA and post hoc Tukey test. The level of significance was set at p ≤ 0.05.

## Results

The comparisons were made using the one-way ANOVA and post hoc Tukey test, a non-parametric test that indicated that there are significant differences between the different groups and subgroups in the study. The study performs an intragroup comparison of CFU counts across different spacer materials and intracanal medicaments after one week (Table 1). Statistically significant differences (p < 0.05) were observed in the cotton (p = 0.045), PTFE (p = 0.049), and endo foam (p = 0.047) groups, indicating variations in microbial contamination. Among subgroups, cotton + MTAP showed the lowest CFU count, while PTFE and endo foam spacers combined with medicaments also demonstrated microbial reduction. These findings highlight the influence of spacer materials and intracanal medicaments on bacterial control, suggesting that some combinations provide better microbial inhibition than others. 

**Table 1:** Intragroup comparison of the CFUs of the groups at the one week interval A one-way ANOVA test was employed; F-values represent the results of ANOVA analysis comparing means among the groups; p < 0.05 was considered statistically significant. The asterisk (*) indicates statistically significant findings. CFU: colony-forming units; Ca(OH)₂: calcium hydroxide; MTAP: modified triple antibiotic paste; PTFE: polytetrafluoroethylene

Group	1-10^3 ^ CFU	10^3^-10^4 ^ CFU	10^4 ^ CFU	p-value	F-value
IA (cotton)	2	2	6	0.045*	2.145
IB (cotton + Ca(OH)_2_)	4	4	2		
IC (cotton + MTAP)	8	2	0		
IIA (PTFE)	6	2	2	0.049*	2.185
IIB (PTFE + Ca(OH)_2_)	2	4	4		
IIC (PTFE + MTAP)	4	4	2		
IIIA (endo foam)	4	2	4	0.047*	1.971
IIIB (endo foam + Ca(OH)_2_)	4	4	2		
IIIC (endo foam + MTAP)	6	2	2		

The study also presents an intergroup comparison of CFU counts at a one-week interval using the post hoc Tukey test (Table 2), highlighting statistically significant differences (p < 0.05) between specific groups. Cotton (IA) vs. PTFE (IIA) (p = 0.041) showed a significant difference, indicating PTFE's superior bacterial control. Cotton + Ca(OH)₂ (IB) vs. PTFE + Ca(OH)₂ (IIB) (p = 0.046) and IIB vs. endo foam + Ca(OH)₂ (IIIB) (p = 0.048) demonstrated significant variation, suggesting PTFE with Ca(OH)₂ may be more effective than cotton. Cotton + MTAP (IC) vs. PTFE + MTAP (IIC) (p = 0.042) and IIC vs. endo foam + MTAP (IIIC) (p = 0.047) further indicate that material selection influences microbial reduction. These results confirm that PTFE and endo foam spacers with intracanal medicaments may enhance bacterial control compared to cotton.

**Table 2:** Intergroup comparison of CFUs at the one week interval The post hoc Tukey test was employed. Post hoc values represent p-values from Tukey’s Honest Significant Difference (HSD) test following ANOVA; p<0.05 was considered statistically significant. The asterisk (*) indicates statistically significant findings. IA: cotton; IIA: PTFE; IIIA: endo foam; IB: cotton + Ca(OH)_2_; IIB: PTFE + Ca(OH)_2_; IIIB: endo foam + Ca(OH)2); IC: cotton + MTAP; IIC: PTFE + MTAP; IIIC: endo foam + MTAP; CFU: colony forming units; Ca(OH)_2_: calcium hydroxide; MTAP: modified triple antibiotic paste; PTFE: polytetrafluoroethylene

Comparision	Mean difference	p-value (post hoc value )
IA vs. IIA	10^4^	0.041*
IA vs. IIIA	10^3^	0.095
IIA vs. IIIA	10^3^	0.093
IB vs. IIB	10^4^	0.046*
IB vs. IIIB	10^3^	0.093
IIB vs. IIIB	10^4^	0.048*
IC vs. IIC	10^4^	0.042*
IC vs. IIIC	10^3^	0.09
IIC vs. IIIC	10^4^	0.04*

## Discussion

The primary goal of endodontic treatment is to eliminate all bacterial presence within the tooth and maintain its sterile condition by preventing further bacterial contamination during and after the treatment [12]. Recent in vitro research has shown that a few days of saliva contact with the coronal root canal filling caused significant coronal leakage. The second most common cause of persistent discomfort after treatment initiation was inadequate temporary restorations during endodontic therapy [13]. To prevent these, 3mm to 4 mm of temporary restorative material is being used along with a material called an endodontic spacer positioned underneath it [14].

Cotton is the most frequently used spacer material placed beneath temporary restorations. Nevertheless, its use poses certain drawbacks, including the potential reduction in the thickness of temporary restorative materials, which should ideally range between 3 mm and 4 mm. The cotton pellet's yielding properties could cause the restorative material on top to shift under masticatory force, jeopardizing the integrity of the pellet. Additionally, cotton's organic and fibrous qualities raise concerns since they may encourage wicking and bacterial absorption [6].

These drawbacks of cotton have led to the use of other materials like foam pellets and PTFE tape as spacers by clinicians. PTFE has been increasingly used as an endodontic spacer due to its advantages similar to those of cotton, with the added potential to address issues like inadequate stiffness and susceptibility to microbial growth. By virtue of its relative inertness, PTFE can withstand acids and solvents and will not break down when combined with dental etchants. PTFE tape, with a melt viscosity nearly six times higher than that of other fluoropolymers, can be sterilized in an autoclave for use in dental applications. Due to these advantages, PTFE tape performed better than cotton in our study and is in accordance with previous studies conducted by Prabhakar et al. [7] and Olsson et al. [15].

Various materials, including foam pellets, have been explored by different clinicians for use as spacers in endodontic treatments [16-18]. These endodontic sponges also serve as convenient tools for chairside storage and mechanical cleaning of root canal instruments. When nickel-titanium (NiTi) rotary instruments are placed within these sponges, they can be effectively cleaned with or without removing their rubber stoppers. However, there aren't many clinical investigations that have examined its use as an endodontic spacer. 

The present study conducted a microbiological evaluation of cotton, PTFE tape, and endo foam used as endodontic spacer materials. The findings revealed a statistically significant difference between the baseline and seven-day mean values across all three groups. This indicates that microbial load increased over the seven-day period in each group. The selection of the seven-day evaluation period was guided by evidence from previous in vivo and in vitro studies [4].

There is no statistically significant difference between cotton and endo foam. After being impregnated with medication, cotton pellets were less conducive to bacterial development. PTFE tape did not absorb the medicament as efficiently due to its polymeric, non-fibrous nature. Endo foam may work better as a spacer material than PTFE if it is sufficiently saturated with medications. But when placed over a spongy spacer, temporary restorations are more likely to break down due to masticatory stresses. Also, the present study demonstrated that bacterial colony formation was less with MTAP than with Ca(OH)₂. Compared to Ca(OH)₂, triple antibiotic paste, which is a combination of three antibiotics, is more effective at treating polymicrobial infections. Ca(OH)₂ due to the release and diffusion of its hydroxyl (OH⁻) ions leads to a highly alkaline environment, which is not conducive to the survival of microorganisms in the root canal [10].

Another critical consideration is that both PTFE and foam do not adhere to the walls of the access cavity, making them easier to place and remove compared to cotton, which often leaves fibers behind and can be more challenging to retrieve. Due to its low kinetic and static coefficients of friction, PTFE facilitates a "non-sticky" application and removal, preventing any residue from remaining within the access chamber [19].

Limitations of the study

The sample size of this study is relatively small. Each endodontic spacer has a unique set of study participants. Every participant will have a distinct level of microbes in their oral cavity. If the split-mouth approach had been used for this study, it might have been more effective.

## Conclusions

Considering the limitations of this study, it can be concluded that PTFE tape and endo foam demonstrated superior performance compared to cotton when used alone as endodontic spacer materials. Therefore, PTFE and endo foam are recommended over cotton in such cases. However, when combined with MTAP, cotton outperformed both endo foam and PTFE tape. These results highlight the critical role of selecting suitable spacer materials to enhance the success of endodontic treatments, improve patient outcomes, and minimize the risk of reinfection.

## References

[1] Shah N, Pisal N, Dedania M, Bajpai MS, Namrata A; Gandhi N (2021). Microbiologic evaluation of cotton, polytetrafluoroethylene tape, and foam as an endodontic spacer material in permanent premolars and molars: an ex vivo study. Endod.

[2] Schwartz RS, Fransman R (2005). Adhesive dentistry and endodontics: materials, clinical strategies and procedures for restoration of access cavities: a review. J Endod.

[3] Mathew A, Lee S, Ha W, Nagendrababu V, Rossi-Fedele G (2021). Microbial contamination comparison between cotton pellet and polytetrafluoroethylene tape endodontic spacers: a systematic review. Eur Endod J.

[4] Shetty A, Garg P, Hegde MN, Rao LN, Shetty C, Shetty S (2019). Microbiological evaluation of polytetrafluoroethylene (PTFE) tape, cellulose sponge and cotton as spacer materials combined with intracanal medicament-an in vitro study. Indian J Public Health Res Dev.

[5] Webber RT, del Rio CE, Brady JM, Segall RO (1978). Sealing quality of a temporary filling material. Oral Surg Oral Med Oral Pathol.

[6] Vail MM, Steffel CL (2006). Preference of temporary restorations and spacers: a survey of Diplomates of the American Board of Endodontists. J Endod.

[7] Prabhakar AR, Dixit K, Raju OS (2018). Microbiologic evaluation of cotton and polytetrafluoroethylene (PTFE) tape as endodontic spacer materials in primary molars an in vivo study. J Clin Pediatr Dent.

[8] Sattar MM, Patel M, Alani A (2017). Clinical applications of polytetrafluoroethylene (PTFE) tape in restorative dentistry. Br Dent J.

[9] (2025). RC-Cal. https://www.prime-dental.in/pdf/rc-cal-literature.pdf.

[10] Parashar V, Khan SA, Singh P, Sharma S, Kumar A, Kumar A (2020). Effect of intracanal medicaments (modified triple antibiotic paste, calcium hydroxide, and Aloe vera) on microhardness of root dentine: an in vitro study. J Contemp Dent Pract.

[11] Djouiai B, Wolf TG (2021). Tooth and temporary filling material fractures caused by Cavit, Cavit W and Coltosol F: an in vitro study. BMC Oral Health.

[12] Jensen AL, Abbott PV, Castro Salgado J (2007). Interim and temporary restoration of teeth during endodontic treatment. Aust Dent J.

[13] Naoum HJ, Chandler NP (2002). Temporization for endodontics. Int Endod J.

[14] Sivakumar JS, Suresh Kumar BN, Shyamala PV (2013). Role of provisional restorations in endodontic therapy. J Pharm Bioallied Sci.

[15] Olsson T, Chan D, Johnson JD, Paranjpe A (2017). In-vivo microbiologic evaluation of polytetrafluoroethylene and cotton as endodontic spacer materials. Quintessence Int.

[16] Hubbard TM Jr, Smyth RN, Pelleu GB Jr, Tenca JI (1975). Chairside decontamination of endodontic files. Oral Surg Oral Med Oral Pathol.

[17] Linsuwanont P, Parashos P, Messer HH (2004). Cleaning of rotary nickel-titanium endodontic instruments. Int Endod J.

[18] Popovic J, Gasic J, Zivkovic S, Petrovic A, Radicevic G (2010). Evaluation of biological debris on endodontic instruments after cleaning and sterilization procedures. Int Endod J.

[19] Biswas SK, Vijayan K (1992). Friction and wear of PTFE-a review. Wear.

